# Efficacy and safety of first-line therapy in patients with HER2-positive advanced breast cancer: a network meta-analysis of randomized controlled trials

**DOI:** 10.1007/s00432-023-05530-3

**Published:** 2024-01-20

**Authors:** Junxiao Wang, Yushuai Yu, Qisheng Lin, Jie Zhang, Chuangui Song

**Affiliations:** 1 Department of Breast Surgery, College of Clinical Medicine for Oncology, Fujian Medical University, No.91, Fuma Road, Jin’an District, Fuzhou, Fujian Province China; 2 Breast Surgery Institute, College of Clinical Medicine for Oncology, Fujian Medical University, Fuzhou, Fujian Province China; 3 Department of Thyroid and Breast Surgery, The Second Hospital of Sanming, Sanming City, Fujian Province China; 4 Department of Breast Surgery, Fujian Medical University Union Hospital, No.29, Xin Quan Road, Gulou District, Fuzhou, 350001 Fujian Province China; 5 Breast Surgery Institute, Fujian Medical University, Fuzhou, Fujian Province China

**Keywords:** Breast cancer, Target therapy, HER2-positive, Advanced, Network meta-analysis

## Abstract

**Purpose:**

The numerous first-line treatment regimens for human epidermal growth factor receptor 2 (HER2)-positive advanced breast cancer (ABC) necessitate a comprehensive evaluation to inform clinical decision-making. We conducted a Bayesian network meta-analysis (NMA) to compare the efficacy and safety of different interventions.

**Methods:**

We systematically searched for relevant randomized controlled trials (RCTs) in Pubmed, Embase, Cochrane Library and online abstracts from inception to June 1, 2023. NMA was performed to calculate and analyze progression-free survival (PFS), overall survival (OS), objective response rate (ORR), and adverse events of grade 3 or higher (≥ 3 AEs).

**Results:**

Out of the 10,313 manuscripts retrieved, we included 28 RCTs involving 11,680 patients. Regarding PFS and ORR, the combination of trastuzumab with tyrosine kinase inhibitors (TKIs) was more favorable than dual-targeted therapy. If only using trastuzumab, combination chemotherapy is superior to monochemotherapy in terms of PFS. It is important to note that the addition of anthracycline did not result in improved PFS. For patients with hormone receptor-positive HER2-positive diseases, dual-targeted combined with endocrine therapy showed better benefit in terms of PFS compared to dual-targeted alone, but it did not reach statistical significance. The comprehensive analysis of PFS and ≥ 3 AEs indicates that monochemotherapy combined with dual-targeted therapy still has the optimal balance between efficacy and safety.

**Conclusion:**

Monochemotherapy (Docetaxel) plus dual-target (Trastuzumab and Pertuzumab) therapy remains the optimal choice among all first-line treatment options for ABC. The combination of trastuzumab with TKIs (Pyrotinib) demonstrated a significant improvement in PFS and ORR, but further data are warranted to confirm the survival benefit.

**Supplementary Information:**

The online version contains supplementary material available at 10.1007/s00432-023-05530-3.

## Introduction

Human epidermal growth factor receptor 2 (HER2)-positive breast cancer (BC) accounts for 15–20% of all BC cases and is characterized by HER2 overexpression, aggressive tumor growth, and poor prognosis (Slamon et al. 1989). With the advent of targeted therapies, treatment outcomes for patients with HER2-positive BC have significantly improved, resulting in a 7-year disease-free survival rate of 93.3% (Romond et al. 2005; Tolaney et al. 2019). Despite standardized treatments, metastasis still occurs in 20–30% of patients with HER2-positive BC (Bear et al. 2015).

Advanced breast cancer (ABC), including both locally advanced breast cancer and metastatic breast cancer, represents a specific subgroup of breast cancer that is resistant to curative surgical interventions. Docetaxel combination with trastuzumab and pertuzumab used in CLEOPATRA trial defines the classic first-line treatment regimen for ABC (Giordano et al. 2022). However, the continuous emergence of drugs is gradually reshaping the treatment landscape, these varying combinations targeted agents offer diverse benefits. For instances, the PHILA study (Xu et al. 2022), the introduction marked a significant departure from the established dual-targeted treatment paradigm for advanced settings, offering new promise for first-line therapy. This study showcased the potential of a trastuzumab and tyrosine kinase inhibitors (TKIs) combination regimen, which demonstrated a numerical advantage over dual-targeted therapy in terms of progression-free survival (PFS). Given the late emergence of pertuzumab, few clinical trials have directly compared it head-to-head with dual-targeted therapy. Consequently, there is a growing need for comprehensive comparisons of all first-line regimens to inform and guide physicians in their choice of therapeutic strategies.

Triple-positive breast cancer (TPBC) characterized as being hormone receptor (HR)-positive and HER2-positive, encompasses various subtypes including Luminal type B and HER2 overexpression type. These subtypes exhibit distinct molecular functions, biological processes, signaling pathways, and clinical behaviors. Furthermore, they demonstrate varying sensitivities to treatment and diverse intrinsic biological features, ultimately reflecting different responses to therapeutic interventions (Zhao et al. 2019). It has thus been proposed that TPBC should be stratified into discrete clinical and genomic subgroups, each potentially warranting a unique treatment approach. Historically, clinical trials of endocrine therapy have predominantly excluded TPBC patients. Similarly, studies focusing on HER2-targeted agents have seldom reported outcomes specifically for the HR-positive subgroup. This has resulted in a paucity of data, leading to low levels of evidence and recommendations for TPBC patients. This area remains controversial and uncertain, underscoring the need for large-scale randomized controlled trials (RCTs).

To address the aforementioned challenges, we propose the application of network meta-analysis (NMA) as a potential solution. NMA is a valuable tool that synthesizes data from multiple clinical studies, thereby enabling indirect comparisons of treatment regimens that have not been compared head-to-head (Li et al. 2011).

Our analysis seeks to identify the optimal regimen for HER2-positive advanced BC, inclusive of the HR-positive subgroup, by delivering a comprehensive and objective assessment of first-line treatment options. Ultimately, our goal is to equip physicians with the evidence necessary to personalize treatment strategies, with the intent of enhancing patient outcomes and quality of life.

## Material and methods

This manuscript follows the Preferred Reporting Items for Systematic Reviews and Meta-Analysis (PRISMA) guidelines (Page et al. 2021).

### Eligibility and Exclusion criteria

We pre-defined the following inclusion and exclusion criteria: (i) phase II or III clinical studies of all HER2-positive ABC undergoing first-line treatment, (ii) RCTs with at least two treatment arms, one of which must contain targeted therapy, (iii) abstracts reported by San Antonio Breast Cancer Symposiums (SABCS), American Society of Clinical Oncology (ASCO), and European Society for Medical Oncology (ESMO), (iv) studies included at least one type of following data: PFS, overall survival (OS), objective response rate (ORR), adverse events of grade 3 or higher (≥ 3 AEs). Single-arm studies, enrollment of less than 10 in either treatment group, retrospective studies, exploratory studies, and clinical studies where first-line data could not be extracted separately, the experimental group was only replaced with a different drug of the same type, the data presented is not detailed enough to be extracted were excluded from our study.

### Information sources

We conducted a comprehensive and detailed search of electronic databases including PubMed, Embase, and Cochrane Library from inception to June 1, 2023. We also performed a search for abstracts published in ASCO, ESMO, and SABCS. References of clinical studies ultimately included in this study were also scanned to avoid omission of other relevant studies, especially for NMA.

### Search strategy

The following search terms was used to retrieved three electronic databases individually: (metastasis OR metastases OR metastatic OR advanced OR recurrent OR stage IV OR unresectable) AND (breast OR mammary) AND (cancer OR carcinoma OR malignant OR neoplasm OR tumour) AND (HER-2 OR HER2 OR HER2/neu OR ERBB2 OR human epidermal growth factor receptor 2) AND (positive OR + OR overexpressing OR overexpressed OR overexpresses) AND (treatment OR therapy OR chemotherapy OR target therapy) AND (randomized controlled trial OR controlled clinical trial OR randomized controlled trial OR double-blind method OR single-blind method OR clinical trial OR clinical trials). The search process uses no filters or restrictions.

### Selection process

Two investigators independently extracted whether these literature met the inclusion criteria. The most recent literature was adopted if multiple papers were generated from the same clinical trial. If doubts about the literature persist, discordance was determinated by a third reviewer. When the abstract does not make it clear that the article fits, the full text needs to be downloaded to evaluate it.

### Data collection process

All data were derived from published data. We extracted data on some of the endpoints present in each study because not every trial contained all the endpoints we required. If the data were not explicitly published, the Engauge Digitizer software was used to calculate the data from the Kaplan–Meier plots.

### Data items

The following data were extracted from the screened literatures: article name, title, the first author, year, treatment, HR status, trial type, tumor stage, the number of participants. The outcomes were analyzed with the following indicators: the primary endpoint PFS, the secondary endpoints OS, ORR, ≥ 3 AEs。

### Study risk of bias assessment

We used Review Manager 5.4 software to assess bias in the involved studies in terms of 6 aspects and 3 levels. The final assessment visualization results were also presented using this software.

### Effect measures

We used the extracted hazard ratio (HazR) values and 95% confidence interval (CI) for PFS and OS calculations. The odd ratio (OR) values and 95% CI were utilized to compare the ORR and ≥ 3 AEs.

### Synthesis methods

After listing each treatment, regimens of the same type were grouped together in the same group, such as those containing two chemotherapeutic agents were grouped in the combination chemotherapy group, pyrotinib or lapatinib were grouped in the TKIs group, and any of the trastuzumab biosimilars were grouped in the biosimilars group. Clinical trials were categorized in the TPBC group for further analysis if the following conditions were met: the enrolled population was HR-positive only; the regimen included endocrine therapy; and data could be extracted from the HR-positive subgroup for analysis.

For PFS and OS, the data were performed using R software. For ORR and ≥ 3 AEs, the data were performed using STATA software. Heterogeneity was assessed using I^2^. We used a random effects model to calculate the results. Surface under the cumulative ranking curve (SUCRA) values ranged from 0 to 100%, higher scores indicate that this regimen is preferred. Finally, we set the maximum values for efficacy (PFS) and safety (≥ 3 AEs) to 50, respectively, and combined the two scores to assess each regimen. *P < *0.05 indicates statistical significance.

### Ethics consideration

This study analyzed published data so ethical consent was not required and all data were derived from published literature. Patients remained anonymous.

## Results

### Study selection

**Fig. 1:**
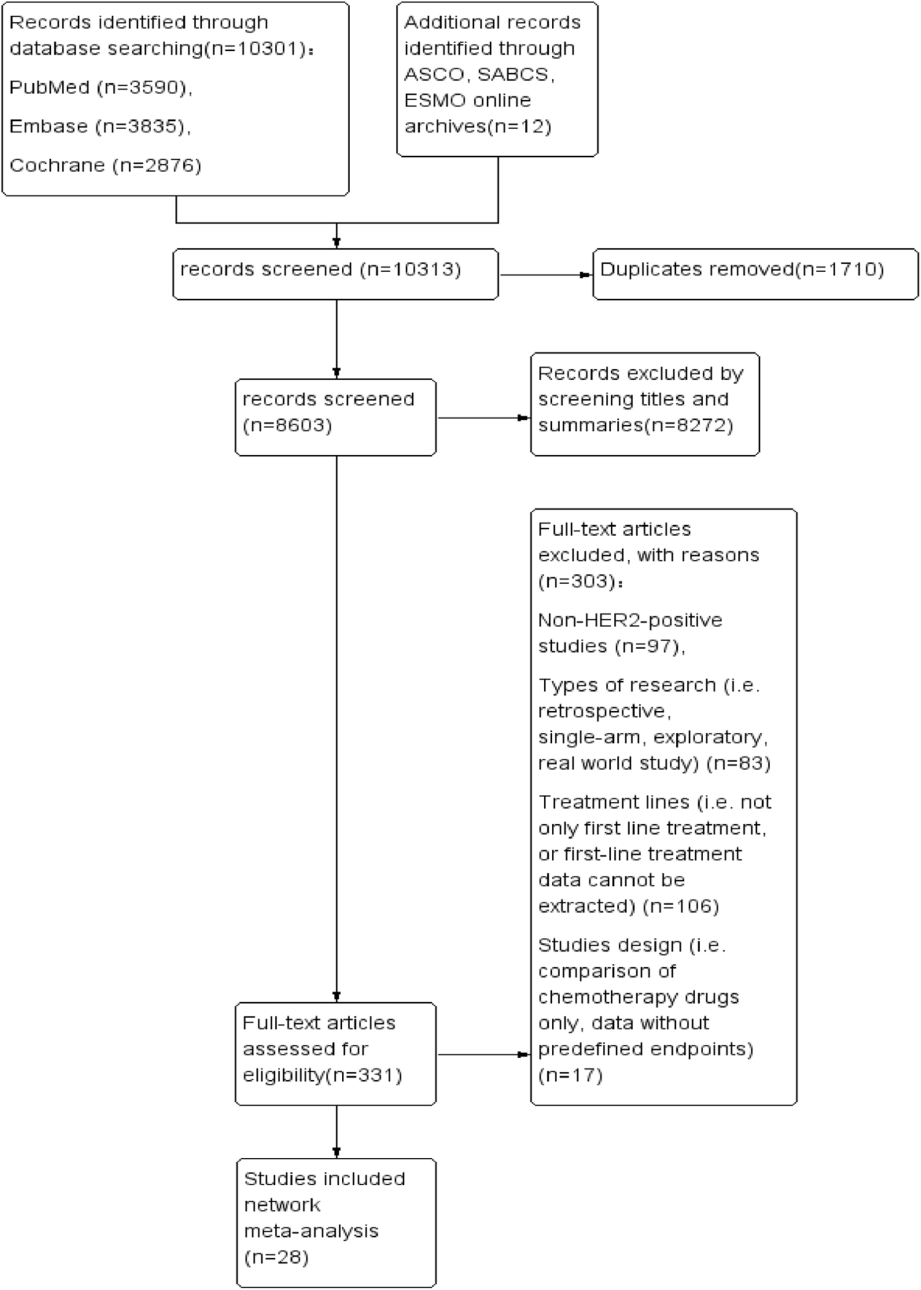
Flowchart of the results of the search and selection process

A total of 10,301 papers were obtained from the database, plus 12 conference abstracts, leaving 8603 papers through the de-duplication process, and then 28 papers containing a total of 11,680 patients were finally screened based on the inclusion and exclusion criteria. We excluded some studies that included multilines therapy because data on first-line therapy could not be extracted separately. Some studies that were only chemotherapy plus target therapy versus another chemotherapy plus target therapy, which did not meet the objectives of our study. PRISMA flow diagram is presented in Fig. 1. PRISMA 2020 item checklist is provided in Supplemental File A.

### Study characteristics

A total of 28 studies from 2001 to 2023 were finally included in the analysis, containing 62 treatment groups and 11,680 patients (Supplemental File B) (Xu et al. 2022, 2023; Pivot et al. 2023; Gianni et al. 2016, 2013; Guan et al. 2013; Rugo et al. 2021; Hurvitz et al. 2015; Swain et al. 2020; Xu et al. 2021a, b; Marty et al. 2005; Rimawi et al. 2018; Huober et al. 2012; Li et al. 2022; Perez et al. 2019, 2014; Valero et al. 2011; Awada et al. 2016; Gelmon et al. 2015; Shao et al. 2020; Baselga et al. 2019; Wardley et al. 2010; Gasparini et al. 2007; Robert et al. 2006; Slamon et al. 2001; Hua et al. 2022; Kaufman et al. 2009; Johnston et al. 2009). Five of the studies included only patients with HER2-positive BC, four were trastuzumab biosimilars, five included dual-targeted regimens, and four included TKIs. All included clinical studies contained one or more prespecified endpoints.

### Risk of bias in studies

**Fig. 2:**
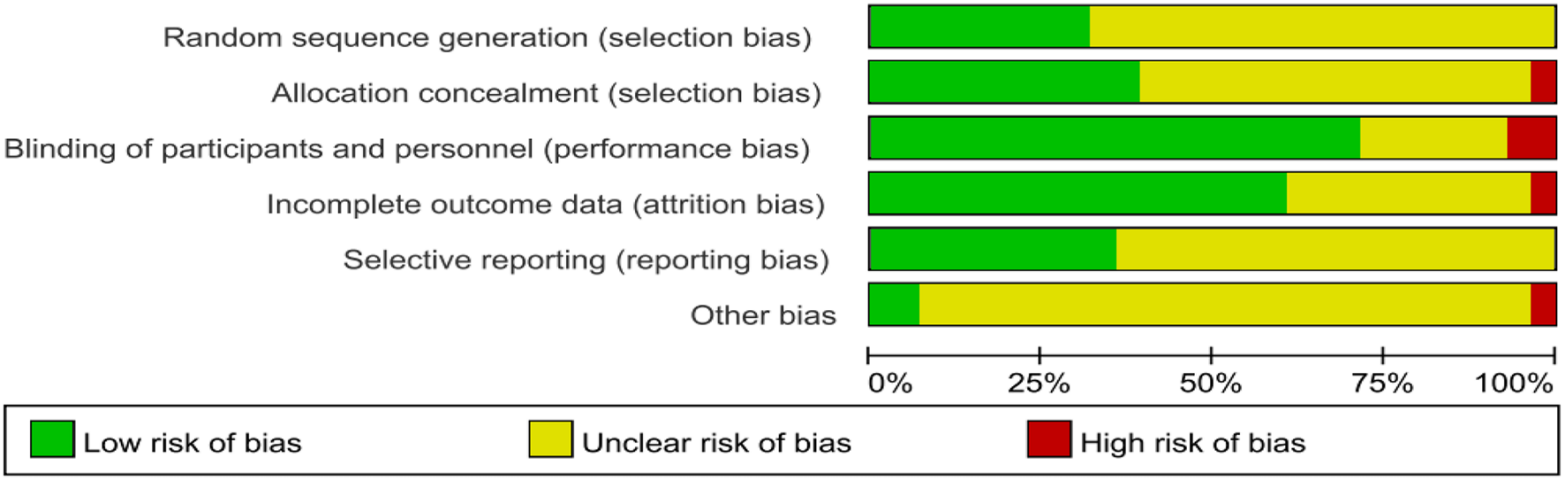
Risk of bias for all enrolled studies

**Fig. 3:**
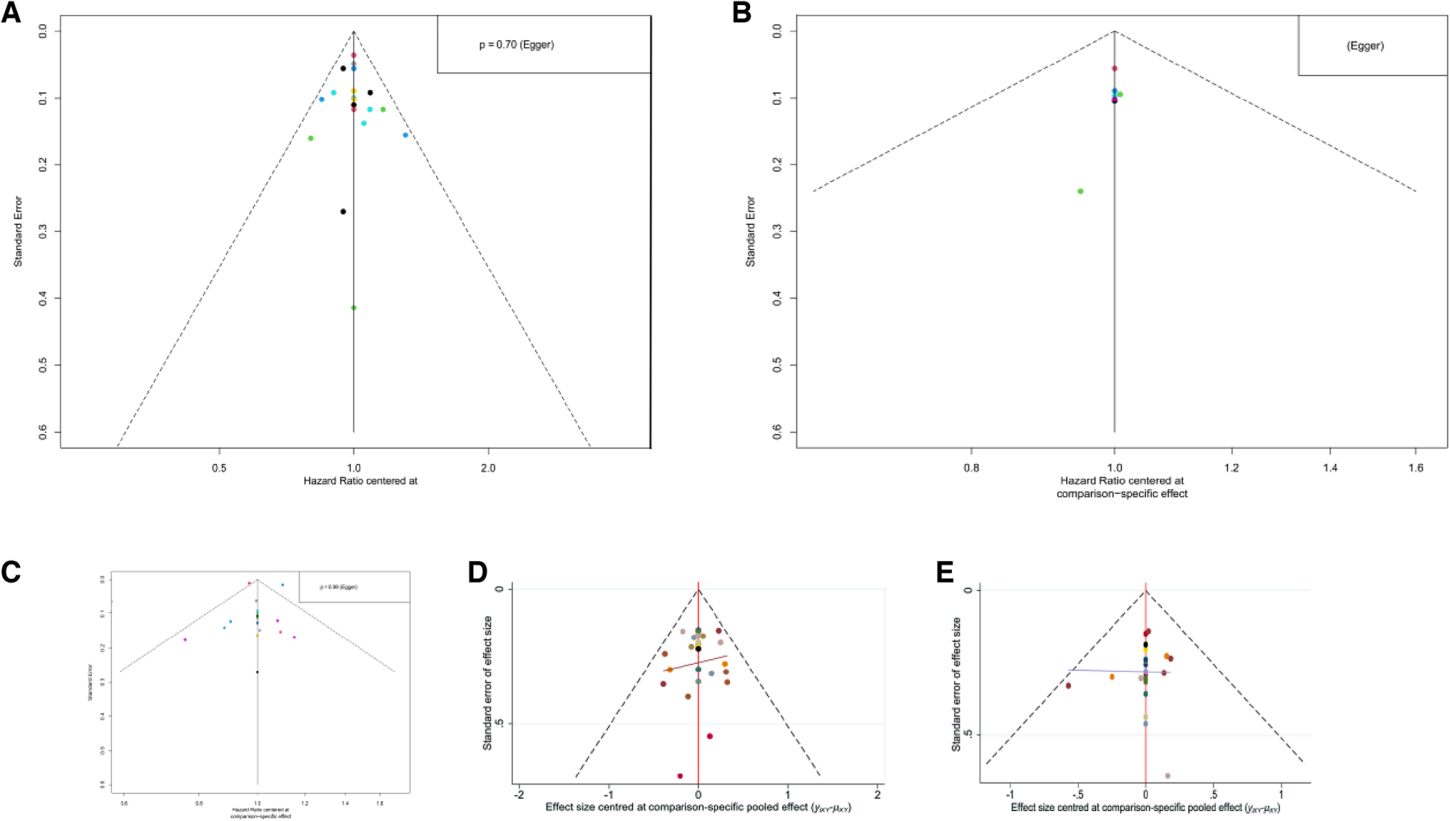
Funnel plots for each predefined endpoint: **A** PFS of overall populations; **B** PFS of HR and HER2 positive populations; **C** OS of overall populations; **D** ORR of overall populations; **E** ≥ 3 AEs of overall populations. Specific details of treatment arms can be found in Fig. 4

According to the risk of bias assessment table, all enrolled trials were at low risk of bias (Fig. 2). Regarding random sequence generation, the randomization method was unknown in 19 studies, but no high risk of bias was reported. Only one study disclosed treatment allocation. Two studies used blinding of participants and personnel, but blinding may be broken, such as early unmasking when the disease progresses or emergency. All of the enrolled studies provided complete outcome data. Ten studies had specific research programs and all of the predefined primary and secondary indicators were reported. One study had potentially biased because the investigator could choose which patients received induction chemotherapy first before random assignment. The funnel plot reveals that there are no publication bias (Fig. 3).

### Primary endpoint PFS

**Fig. 4:**
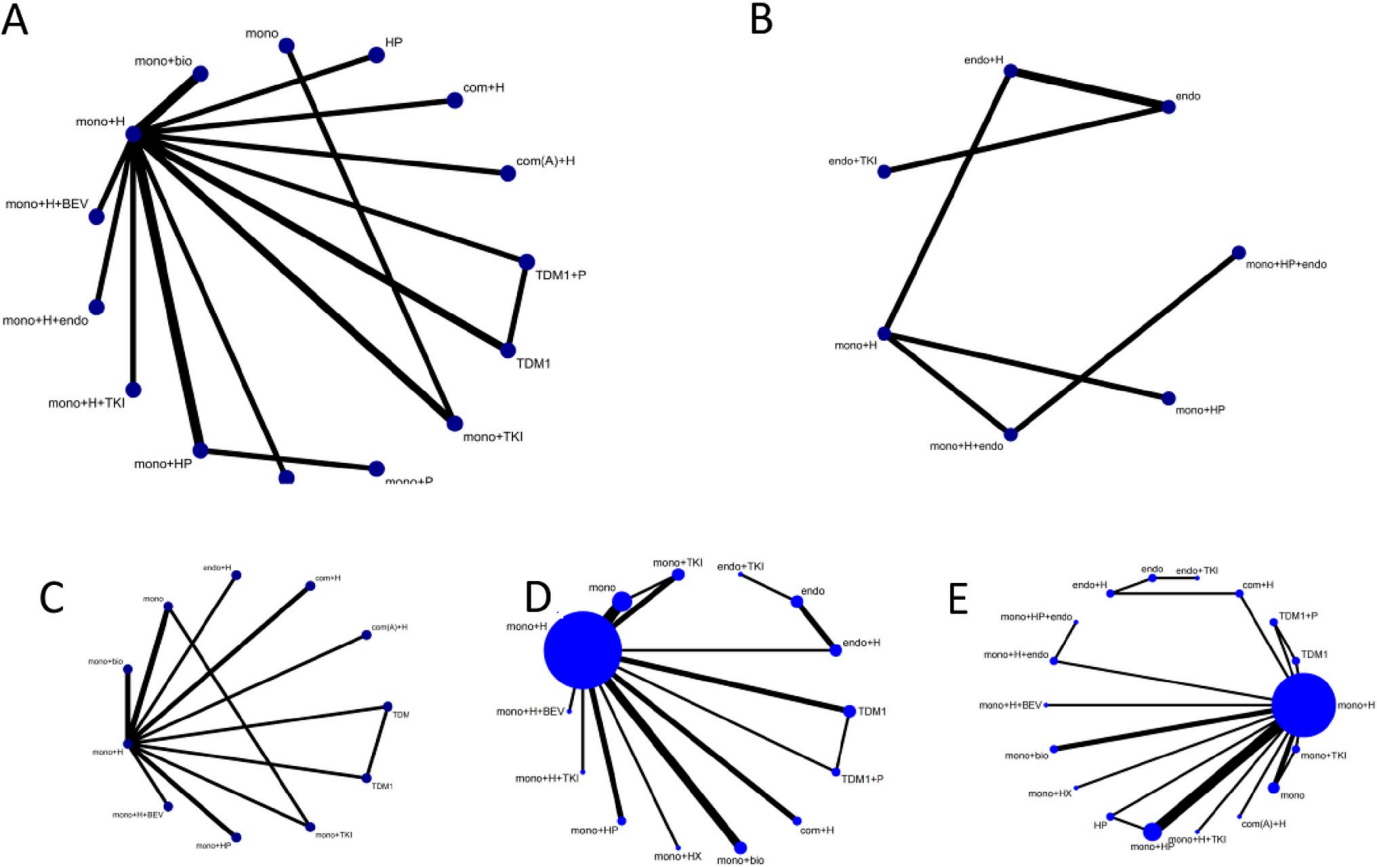
Network diagrams for each predefined endpoint: **A** PFS of overall populations; **B** PFS of HR and HER2 positive populations; **C** OS of overall populations; **D** ORR of overall populations; **E** safety of overall populations. Specific details of treatment arms: Mono, monochemotherapy; Mono + bio, monochemotherapy + trastuzumab biosimilar; Mono + H, monochemotherapy + trastuzumab; Mono + H + BEV, monochemotherapy + trastuzumab + bevacizumab; Mono + H + endo, monochemotherapy + trastuzumab + endocrine therapy; Mono + H + TKI, monochemotherapy + trastuzumab + tyrosine kinase inhibitors (pyrotinib or lapatinib or neratinib); Mono + HP, monochemotherapy + trastuzumab + pertuzumab; Mono + HX, monochemotherapy + trastuzumab + capecitabine; Mono + P, monochemotherapy + pertuzumab; Mono + TKI, monochemotherapy + tyrosine kinase inhibitors (pyrotinib or lapatinib or neratinib); T-DM1, trastuzumab emtansine; TDM1 + P, trastuzumab emtansine + pertuzumab; Com(A) + H, combine chemotherapy (anthracycline included) + trastuzumab; Com + H, combine chemotherapy (without anthracycline) + trastuzumab; HP, trastuzumab + pertuzumab. Endo, endocrine therapy; Endo + H, endocrine therapy + trastuzumab; Endo + TKI, endocrine therapy + tyrosine kinase inhibitors (pyrotinib or lapatinib or neratinib); Mono + HP + endo, monochemotherapy + trastuzumab + pertuzumab + endocrine therapy

**Fig. 5:**
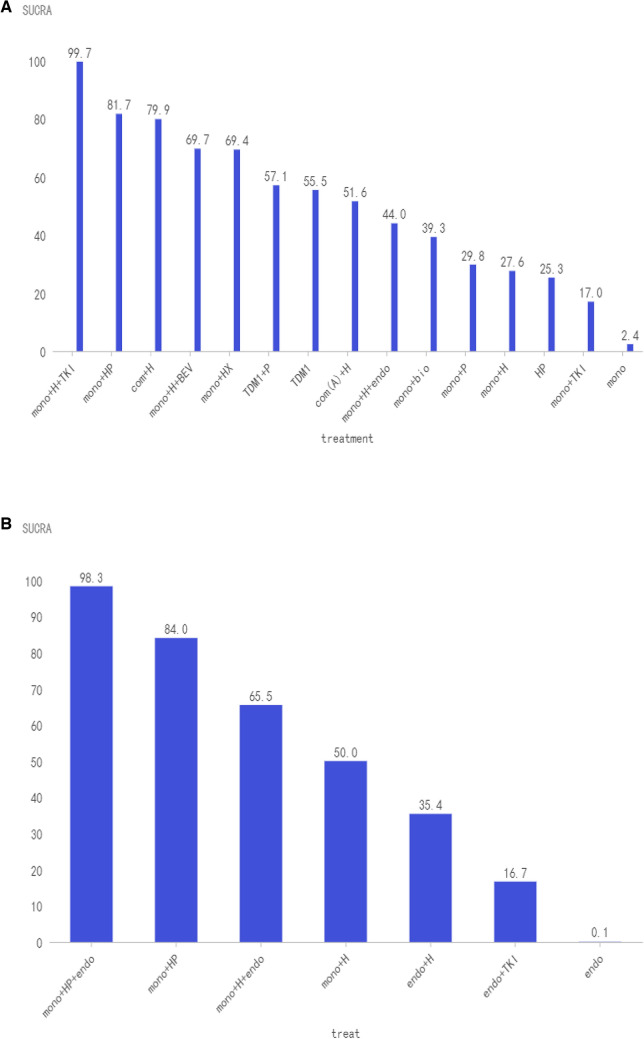

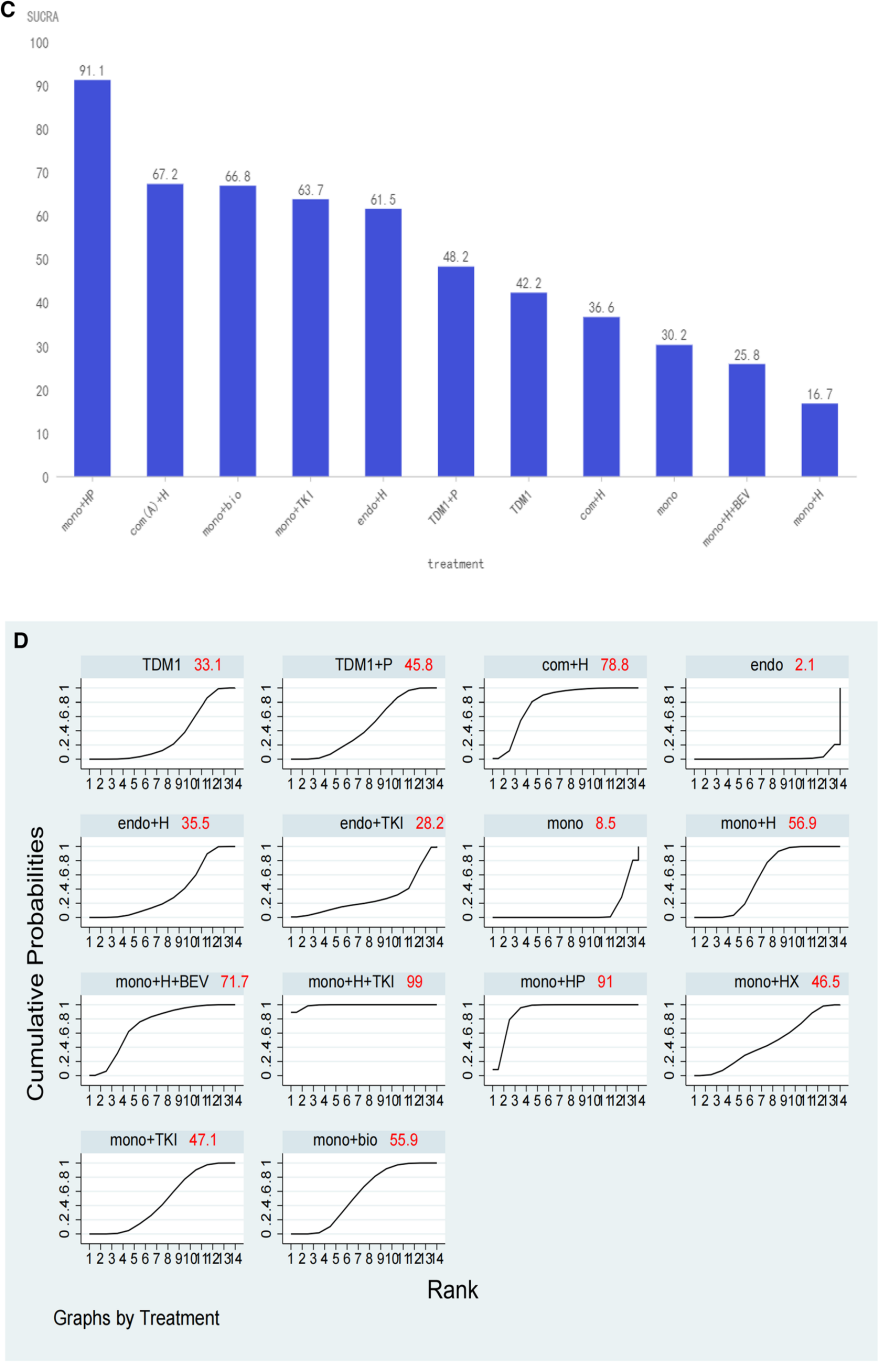

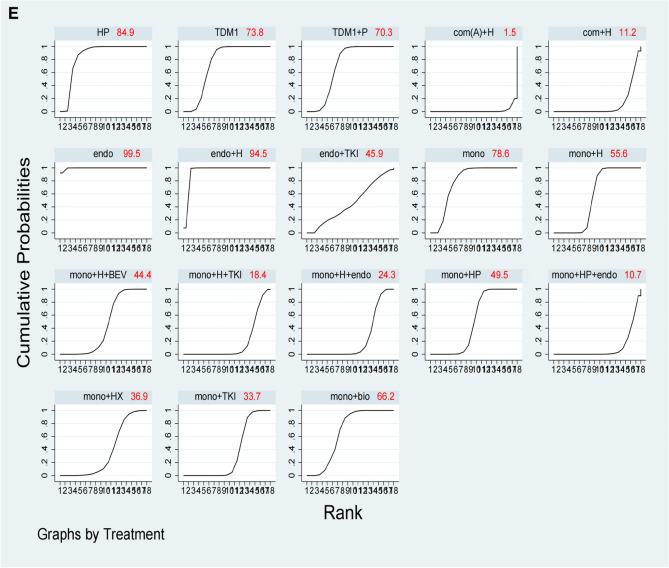
SUCRA values and rankings for each outcome measure: **A** PFS in the overall population; **B** PFS in the HR and HER2 positive population; **C** OS in the overall population; **D** ORR in the overall population; (E) ≥ 3 AEs in the overall population. Specific information on treatment regimens can be found in Fig. 4

Twenty-two of the enrolled studies reported primary endpoints, and specific network diagrams can be found in Fig. 4A. As analyzed by NMA, monochemotherapy in combination with trastuzumab plus TKIs ranked the highest in terms of PFS of SUCRA, followed by monochemotherapy combines trastuzumab plus pertuzumab, and then combination chemotherapy plus trastuzumab. We can see that T-DM1 also had appropriate efficacy but does not rank high. Trastuzumab biosimilars are slightly better than trastuzumab. Monochemotherapy combined with TKIs is less effective (Fig. 5A).

Indirect comparisons are seen in Table 1, 17 studies were cross-compared. Trastuzumab combined with TKIs had a significantly higher PFS than trastuzumab with patuzumab [mono + H + TKI vs mono + HP HazR: 0.54 (95%CI: 0.40–0.72)]. Adding anthracyclines to the regimen does not improve efficacy [com(A) + H vs mono + H HazR: 0.84 (95%CI: 0.62–1.14)]. TKI alone does not work better than dual-targeted [mono + H + TKI vs mono + TKI HazR: 0.3 (95%CI: 0.21–0.41), mono + HP vs mono + TKI HazR: 0.55 (95%CI: 0.41–0.74)]. In China, some patients are still unable to use pertuzumab due to economic reasons and health insurance policy. If only trastuzumab is used, the combination of chemotherapy demonstrates superior PFS compared to monochemotherapy [com + H vs mono + H HazR: 0.66 (95%CI: 0.53–0.83)].

**Table 1:**
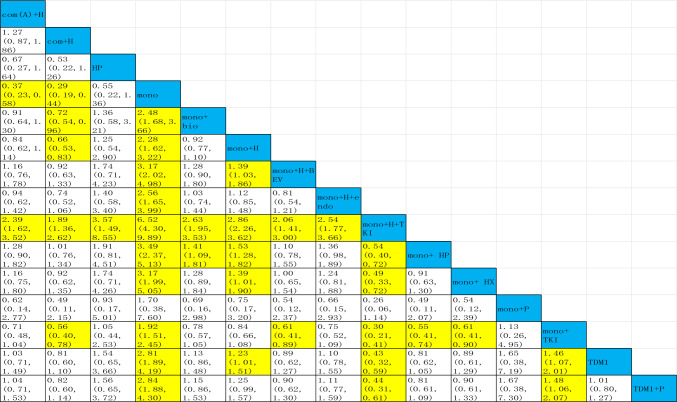
Cross-comparison of PFS for total population

The results after making comparisons of the treatment regimens in each row and column are shown as HazR and 95% CI results, and results with *p < *0.05 are highlighted in yellow. Specific details of treatment arms can be found in Fig. 4

For the TPBC population, the network diagram of PFS and the SUCRA ranking can be presented specifically in Figs. 4B and Fig. 5B. All regimens contain chemotherapy ranked higher than regimens without chemotherapy. The sucra of monochemotherapy + dual-targeted + endocrine therapy ranked the highest, better than monochemotherapy + dual-targeted therapy, but there was no statistical significance in the cross-comparison (Supplemental File C1).

### Secondary endpoint OS

Thirteen studies contained OS data, and the network diagram is shown in Fig. 4C. From the SUCRA rankings, monochemotherapy + dual-targeted therapy was the highest and had a much larger score than the other regimens (Fig. 5C). Single-targeted therapy is far less effective than double-targeted [mono + H vs mono + HP HazR: 1.46 (95%CI: 1.19–1.79)]. The administration of anthracyclines does not improve OS [com(A) + H vs com + H HazR:0.85 (95%CI: 0.60–1.22), com(A) + H vs mono + H HazR:0.79 (95%CI: 0.59–1.07)] (Supplemental File C2).

### Secondary endpoint ORR

Twenty-two studies reporting data on ORR included in final analysis. The network plot is available in Fig. 4D. Monochemotherapy combined with trastuzumab and TKIs was on the top rank in SUCRA, as with PFS, was superior to monochemotherapy plus dual-targeted. Besides that, these two options outperform the others (Fig. 5D). However, in the league table, the combination of monotherapy with trastuzumab and pertuzumab was more effective than the combination of monotherapy with trastuzumab and TKIs [mono + HP vs mono + H + TKI OR: 0.4 (95%CI: − 0.98 to 0.18)], which may be related to the performance of the two treatment regimens in other evaluation indicators (Supplemental File C3).

### Secondary endpoint safety

Toxicity differs from drug to drug, so it is not accurate to assess all regimens with one complication, for instance, TKIs do not have a high level of hematologic toxicity, but the incidence of diarrhea is remarkable. ≥ 3 AEs was ultimately chosen as an index to evaluate safety, as it is what most clinical studies will count, and it has a tremendous impact on the patient's quality of life. Twenty-two studies were analyzed for ≥ 3 AEs, and as with those endpoints above, the network diagram is also displayed in Fig. 4E. Endocrine therapy, trastuzumab, pertuzumab were best tolerated in terms of SUCRA score rankings. It is worth mentioning that T-DM1 has a low rate of ≥ 3 AEs, which may be related to the fact that his thrombocytopenia can be well prevented and controlled. Monochemotherapy plus dual-targeted is ranked much higher than monochemotherapy + H + TKI (Fig. 5E). Dual-targeted has fewer complications than single target plus TKI [mono + HP vs mono + H + TKI OR: − 0.91(95%CI: − 1.42 to 0.40)](Supplemental File C4).

### Combined analysis of PFS and ≥ 3 AEs

**Fig. 6:**
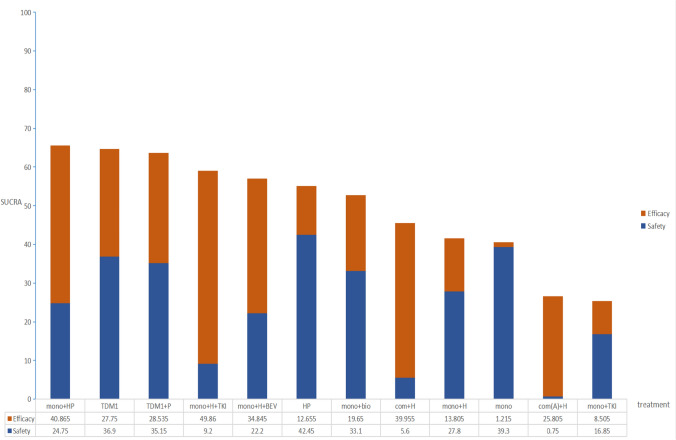
Rankings derived from combining PFS and ≥ 3 AEs. The maximum values of PFS and ≥ 3 AEs are 50 each, and they are added together and ranked in order of score. Specific details of treatment arms can be found in Fig. 4

Regimens that are both effective and safe will definitely become the first choice of patients. monochemotherapy + dual-targeted had the highest ranked SUCRA score, which was significantly higher than monochemotherapy + H + TKI because of the high complications of TKIs. It is interesting to note that T-DM1 is in second place, which may be related to the controllable complications. Regardless of whether anthracycline is included or not, combination chemotherapy plus trastuzumab is ranked poorly because of its high complication (Fig. 6).

## Discussion

Patients with advanced breast cancer often deviate from rigid guidelines and necessitate multidisciplinary treatment teams discussions to incorporate additional factors such as the patient's underlying conditions, economic situation, tolerance to previous treatments, accessibility of medication, patient and family preferences, current guidelines and consensus. Patients have the opportunity to actively engage in their treatment process by gaining a comprehensive understanding of their medical condition, articulating queries and concerns, and expressing their individual preferences for available treatment options. Our research provides rankings for various first-line chemotherapy regimens, which can guide physicians in quickly selecting the most effective and safest option when multiple chemotherapy regimens are available for selection.

There is no doubt that dual-targeted therapy consisting of trastuzumab and pertuzumab is the first choice for first-line treatment of ABC. But with the development of anti-HER2 drugs, this dual-targeted combination may be challenged as never before. Our study showed that TKIs combined with trastuzumab was superior to pertuzumab combined with trastuzumab in some aspects.

Tyrosine kinase inhibitors (TKIs) have demonstrated certain benefits in both neoadjuvant settings and ≥ 2 line treatment in ABC, as evidenced by multiple studies (Wu et al. 2022; Xu et al. 2021a, b). These agents are progressively being considered for first-line treatment as supporting evidence accumulates. In our investigation, the combination of trastuzumab with TKIs yielded the highest PFS SUCRA score (Fig. 5A), significantly surpassing that of the trastuzumab-pertuzumab combination. Prior research has indicated limited effectiveness of TKIs combined with monochemotherapy (Guan et al. 2013; Gelmon et al. 2015), however, the integration of trastuzumab with TKIs appears to produce favorable outcomes. The advantages of TKIs over pertuzumab are in three aspects: 1. TKIs covalently interact with the ATP-binding sites in the intracellular kinase domains of HER1, HER2, and HER4, thus impeding downstream signaling (Li et al. 2017). 2. TKIs can penetrate the central nervous system and have unexpected efficacy in patients with BC brain metastases (Morikawa et al. 2015). In the brain metastasis subgroup of the HER2CLIMB study, the 1-year PFS was much higher in the tucatinib group than in the control group (24.9% vs 0%, HazR 0.48; 95%CI 0.34–0.69; *p < *0.001)(Murthy et al. 2020). 3. TKIs in combination with trastuzumab can comprehensively block both intracellular and extracellular domains and significantly improve anti-HER2 treatment efficacy, which has been demonstrated in the treatment of early-stage BC (Wu et al. 2022; Llombart-Cussac et al. 2017).

Our analysis also revealed that the combination of trastuzumab and TKIs had the highest SUCRA value in terms of ORR, which is consistent with the data from the PHILA study that reported an ORR of 88.2% (Xu et al. 2022). This exceeded the ORR of 80.3% reported in the CLEOPATRA study (Swain et al. 2020), indicating that this treatment regimen has a certain efficacy in promoting tumor shrinkage. ≥ 3 AEs are generally intolerable for patients, necessitating a careful balance between treatment efficacy and safety. A synthesized analysis of PFS and ≥ 3 AEs serves as an optimal guide for clinicians in selecting a treatment regimen. Trastuzumab combined with TKIs, despite ranking second of PFS (Fig. 5A), exhibited a substantial rate of ≥ 3 AEs (Fig. 5E), that's why the overall ranking is not high (Fig. 6). Diarrhea identified as a notable complication. The PANDORA study (Wang and Huang 2022) demonstrated that the administration of granulocyte colony-stimulating factor and loperamide, following a protocol modification, effectively mitigated complications, enhancing patients’ treatment tolerance. Consequently, TKIs may represent a promising future therapeutic option.

Our study suggests that T-DM1 has a moderate PFS (Fig. 5A), but he has a low complication rate (Fig. 5E), so after combining PFS and ≥ 3 AEs scores, it is second only to the current standard monochemotherapy combination of dual-targeted therapy in terms of recommendation (Fig. 6). T-DM1, representing the inaugural antibody–drug conjugate (ADC) combination of monochemotherapy combination with targeted therapy, has been investigated as a first-line treatment option for advanced breast cancer (ABC). The MARIANNE trial (Perez et al. 2019) and the TDM4450g study (Perez et al. 2014) suggest that first-line treatment with T-DM1 yields comparable results to monochemotherapy in combination with trastuzumab in terms of PFS, so it a viable alternative for patients who are unable to tolerate dual-targeted regimens. It is important to highlight that trastuzumab deruxtecan (T-Dxd), recognized for its substantial effectiveness in the treatment of advanced disease beyond the second line (Hurvitz et al. 2023), is a coupling of trastuzumab and deruxtecan and has a higher drug-to-antibody ratio than T-DM1 (8:3.5) (Doi et al. 2017). The bystander effect is another of its features, and chemotherapeutic drugs released after being endocytosed can diffuse to neighboring cells, helping to overcome HER2 heterogeneity. Currently under investigation as a first-line treatment option, and the oncology community eagerly awaits the release of these data (Tolaney et al. 2022).

TPBC represents a distinct clinical entity. While HER2 positivity is generally an independent predictor of poor prognosis, high HR expression is associated with a better outcome (Parise and Caggiano 2016; Kay et al. 2021). Thus, patients with HR-positive/HER2-positive status tend to have a better prognosis than their HR-negative/HER2-positive counterparts but fare worse than HR-positive/HER2-negative patients. Notably, crosstalk between the HR and HER2 pathways complicates treatment, as monochemotherapy targeting either pathway alone can lead to suboptimal results and potential drug resistance. For example, the CLEOPATRA study (Swain et al. 2020) showed reduced benefits from dual-targeted chemotherapy in HR-positive patients compared to HR-negative patients. The BIG 1–98 study (Rasmussen et al. 2008) reported poorer outcomes for patients with HER2-positive BC treated with endocrine therapy alone. Our analysis indicates that combining dual-targeted therapy with endocrine therapy yields the highest SUCRA of PFS for TPBC patients, outperforming both monochemotherapy plus dual-targeted and monochemotherapy plus single-targeted and endocrine therapy (Fig. 5B). Although this recommendation is based on data from the phase II PERTAIN trial (Rimawi et al. 2018) alone, it is supported by findings from the MonarcHER (André et al. 2022) and SYSUCC-002 (Hua et al. 2022) studies, suggesting that this combined approach represents a promising and effective strategy for managing TPBC.

For patients with cardiac comorbidity or risk factors for heart disease, cardiac function testing is required to exclude contraindications for trastuzumab and anthracycline drugs. Our research has found that T-DM1, which has no significant cardiac toxicity, is a good alternative for such patients (Fig. 6).

While improved PFS is a notable outcome, it is not a definitive marker of treatment effectiveness unless it translates into a benefit in OS (Adunlin et al. 2015). Unfortunately, only the regimen of monochemotherapy plus dual-targeted therapy is currently benefiting on the OS (Supplemental File D3). We acknowledge that the OS data in many of these studies remain immature due to the lengthy process of OS data collection. As a result, only OS results from a small number of studies were included in our current analysis. We anticipate future research that comprehensively evaluates treatment options from the perspective of OS.

## Limitations

All of our studies enrolled RCTs to improve accuracy and reliability and utilized PFS, OS, ORR and ≥ 3 AEs to comprehensively assess the efficacy and safety of the regimens. It was also analyzed for the triple-positive subgroup. But our study still have some shortcomings: we excluded single-arm studies to form a closed loop; Some studies did not publish information on randomization and allocation methods, which can lead to potential bias; Some studies did not include all endpoints, or the endpoints were immature, and our analysis based on existing published data would have resulted in potential bias; Some studies enrolled patients on first and second line treatments, and it was not possible to extract data on first-line treatments separately.

## Conclusion

This NMA utilizing Bayesian modeling provides valuable insights into the first-line treatment options for HER2-positive ABC. Monochemotherapy (Docetaxel) plus dual-target (Trastuzumab and Pertuzumab) therapy emerged as the optimal choice based on their efficacy outcomes of OS and a comprehensive analysis of both PFS and ≥ 3 AEs. Additionally, the combination of trastuzumab with TKIs (Pyrotinib) offered a more favorable PFS and ORR, but further data are warranted to confirm the survival benefit.

### Supplementary Information

Below is the link to the electronic supplementary material.

Supplementary file1 (PDF 45 kb)

Supplementary file2 (XLSX 21 kb)

Supplementary file3 (DOCX 189 kb)

Supplementary file4 (DOCX 342 kb)

## Data Availability

All data generated or analyzed during this study are included in the supplemental file of this published article.

## Abbreviations

HER2: Human epidermal growth factor receptor 2
BC: Breast cancer
ABC: Advanced breast cancer
TKIs: Tyrosine kinase inhibitors
PFS: Progression-free survival
TPBC: Triple-positive breast cancer
HR: Hormone receptor
RCTs: Randomized controlled trials
NMA: Network meta-analysis
PRISMA: Preferred Reporting Items for Systematic reviews and Meta-analyses
SABCS: San Antonio Breast Cancer Symposium
ASCO: American Society of Clinical Oncology
ESMO: European Society for Medical Oncology
OS: Overall survival
ORR: Objective response rate
≥ 3 AEs: Adverse events of grade 3 or higher
HazR: Hazard ratio
CI: Confidence Interval
OR: Odd ratio
SUCRA: Surface under the cumulative ranking curve
ADC: Antibody-drug conjugate
