# Supplementary material for: Efficacy and safety of first-line therapy in patients with HER2-positive advanced breast cancer: a network meta-analysis of randomized controlled trials
Source: J Cancer Res Clin Oncol. 2024 Jan 20;150(1):21. doi: 10.1007/s00432-023-05530-3 (PMC10799814; doi:10.1007/s00432-023-05530-3)
Supplement: Supplementary file 3 — Supplementary file3 (DOCX 189 kb) [file 432_2023_5530_MOESM3_ESM.docx]

1

2

3

4

Supplemental file C: Cross-comparison of each endpoints. (1) PFS of HR+HER2+ population. The results after making comparisons of the treatment regimens in each row and column are shown as HR and 95% CI results, and results with p < 0.05 are highlighted in yellow. (2) OS of overall populations. The results after making comparisons of the treatment regimens in each row and column are shown as HR and 95% CI results, and results with p < 0.05 are highlighted in yellow. (3) ORR of overall populations. The results after making comparisons of the treatment regimens in each row and column are shown as OR and 95% CI results, and results with p < 0.05 are highlighted in yellow. (4) Satety of overall populations. The results after making comparisons of the treatment regimens in each row and column are shown as OR and 95% CI results, and results with p < 0.05 are highlighted in yellow.
