# Supplementary material for: Efficacy and safety of first-line therapy in patients with HER2-positive advanced breast cancer: a network meta-analysis of randomized controlled trials
Source: J Cancer Res Clin Oncol. 2024 Jan 20;150(1):21. doi: 10.1007/s00432-023-05530-3 (PMC10799814; doi:10.1007/s00432-023-05530-3)
Supplement: Supplementary file 4 — Supplementary file4 (DOCX 342 kb) [file 432_2023_5530_MOESM4_ESM.docx]

1
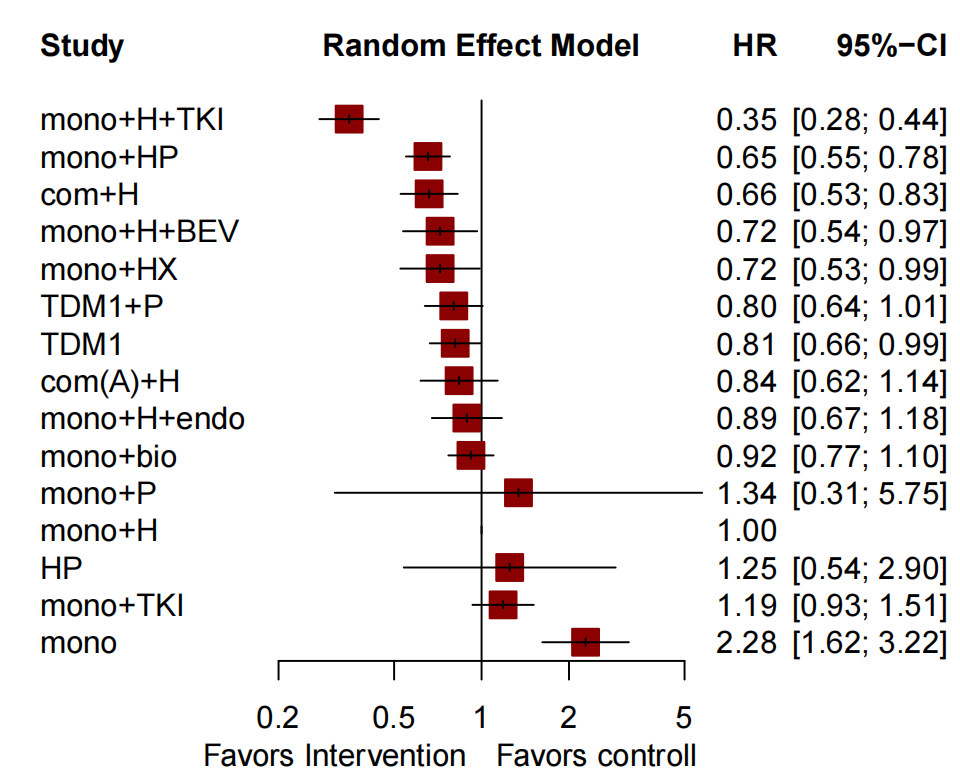


2
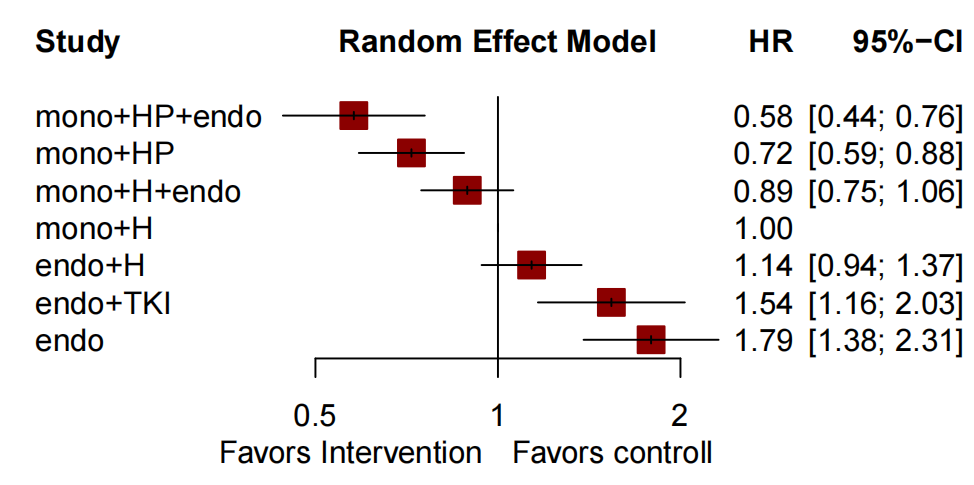


3
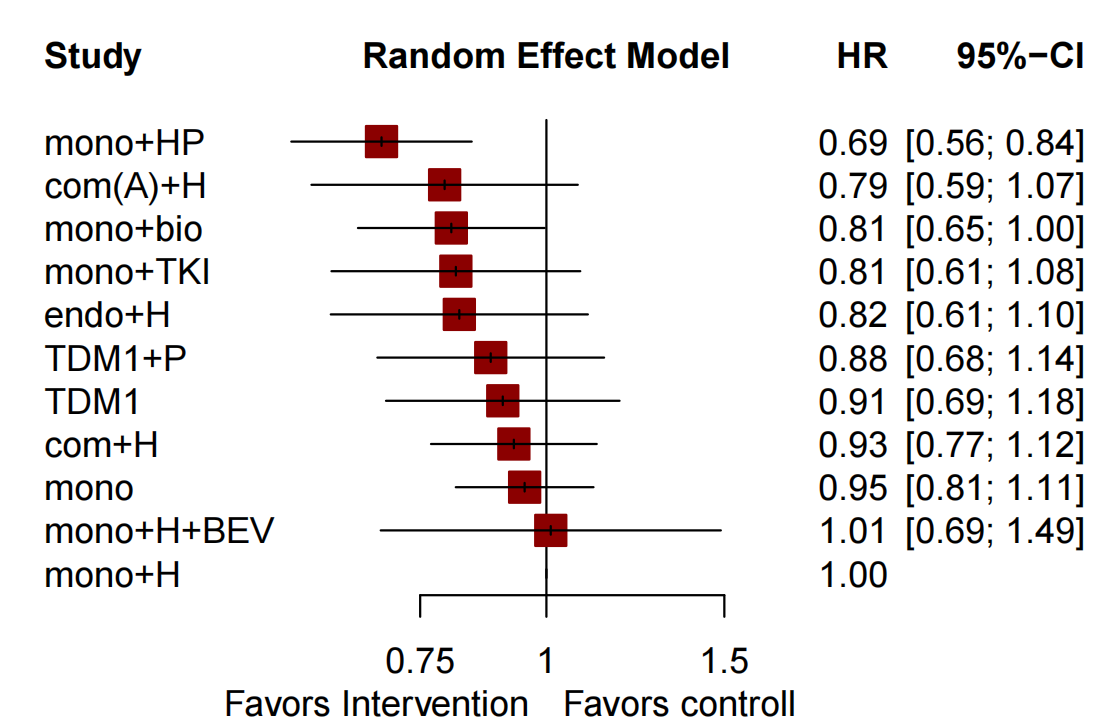


Supplemental file D: Forest plot of each endpoints. (1) PFS of total population. (2) PFS of HR+HER2+ population. (3) OS of total population.
